# The effects of aging and perceived loneliness on lexical ambiguity resolution

**DOI:** 10.3389/fpsyg.2022.978616

**Published:** 2022-10-20

**Authors:** Nannan Zhou, Chih-Mao Huang, Qing Cai, Ovid J. L. Tzeng, Hsu-Wen Huang

**Affiliations:** ^1^Institute of Brain and Education Innovation, School of Psychology and Cognitive Science, East China Normal University, Shanghai, China; ^2^Shanghai Center for Brain Science and Brain-Inspired Technology, Shanghai, China; ^3^Department of Biological Science and Technology, National Yang Ming Chiao Tung University, Hsinchu, Taiwan; ^4^Center for Intelligent Drug Systems and Smart Bio-Devices (IDS^2^B), National Yang Ming Chiao Tung University, Hsinchu, Taiwan; ^5^Cognitive Neuroscience Laboratory Institute of Linguistics, Academia Sinica, Taipei, Taiwan; ^6^College of Humanities and Social Sciences, Taipei Medical University, Taipei, Taiwan; ^7^Department of Educational Psychology and Counseling, National Taiwan Normal University, Taipei, Taiwan; ^8^Hong Kong Institute for Advanced Study, City University of Hong Kong, Kowloon, Hong Kong SAR, China; ^9^Department of Linguistics and Translation, City University of Hong Kong, Kowloon, Hong Kong SAR, China

**Keywords:** aging, perceived loneliness, sublexical ambiguity, reading, Chinese compounds

## Abstract

Language is central to the interactional nature of the social life within which it is situated. To react or respond in a particular situation, we must be able to recognize the social situation. Growing evidence has demonstrated the negative impact of perceived loneliness on late-life executive functions. Yet little is known about how social factors impact language processing for older people. The current study aims to fill this gap, first by assessing age-related changes in lexical processing during Chinese word reading, second, by examining whether older adults’ individual differences, such as processing speed and verbal abilities, modulate meaning retrieval and, third, by investigating whether perceived loneliness can hinder word reading. The use of compound words in Chinese enables significant sublexical ambiguity, requiring varying executive load during word recognition: when a word’s constituent characters carry multiple meanings, readers must consider the meaning contributions of both constituent characters and use top-down word information to determine the most accurate meaning of the ambiguous character, a process termed “sublexical ambiguity resolution.” In this study, adults read real Chinese words (including both sublexically ambiguous and unambiguous words) and pseudowords, and they were asked to make lexical decisions. Older adults exhibited greater lexicality effects (i.e., real words were easier to be identified than pseudowords) and similar sublexical ambiguity effects compared with young adults. Among older participants, processing speed could account for their ability to differentiate between words and pseudowords. In contrast, the level of perceived loneliness modulated the efficacy of sublexical ambiguity resolution: the participants with higher perceived loneliness displayed a greater sublexical ambiguity disadvantage effect. These results indicate that perceived loneliness may affect the use of contextual information in meaning retrieval during reading. The findings provide an important link between social connections and language processing.

## Introduction

As a social species, humans rely on a safe, secure social environment to survive and thrive. Language plays a prominent role in social interactions because we communicate our thoughts and feelings to others with language. Reading, as one of the language skills, not only has a great impact on communication and learning but is also one of the most appreciated leisure activities of older adults—one that could help them maintain functional independence. Therefore, reading ability has a significant impact on quality of life for older people.

Reading is a multifaceted process that spans sensory pattern recognition, memory access and attention control for meaning selection. As such, reading depends both on crystallized semantic intelligence that grows or is maintained through healthy aging, and on components of fluid intelligence such as information transformation and attention control that decline with age (Stine-Morrow, 2007). In line with this, aspects of reading that mainly rely on word-level knowledge, such as word associations and semantic priming, have been shown to remain stable with age (e.g., Burke and Peters, 1986; Burke et al., 1987). In contrast, more demanding processes, such as rapidly integrating lexical information to form coherent representations, have been found to be substantially altered in older adults (Meyer and Federmeier, 2010; Huang et al., 2012).

Ambiguity resolution in word reading involves not only recognizing the word but also recalling multiple potential meanings and eliminating contextually inappropriate interpretations. One orthographic form can be associated with multiple meanings, leading to a one-to-many relationship between a form and its referents. A deficit in ambiguity resolution would therefore be detrimental to daily language tasks, as lexical ambiguity is one of the most distinguishing features of written language (Rodd et al., 2002; Huang C. Y. et al., 2011). Many studies on word recognition in alphabetic languages in young adults have reported that words with multiple unrelated meanings hinder the word recognition process compared with unambiguous words, a phenomenon termed the ambiguity disadvantage effect (Frazier and Rayner, 1990; Klepousniotou, 2002; Rodd et al., 2002; Beretta et al., 2005; Pylkkänen et al., 2006; Klepousniotou et al., 2012). Given the well-recognized decline in older adults’ executive function, one might expect that they would experience increased difficulty when resolving ambiguity compared with younger adults. Indeed, behavioral studies have shown that older adults have increased difficulty inhibiting contextually inappropriate meanings of homonyms (e.g., bank) compared with younger adults (Faust et al., 1997) when ambiguous words are embedded in a sentence. Studies of event-related brain potentials (ERPs) have further demonstrated that older adults are less likely to engage in controlled processes to revise an existing interpretation of ambiguous words to adapt to a change in contextual focus (Meyer and Federmeier, 2010). In Chinese, most words are compounds, and the constituent characters within a word can have different meanings on their own. When a Chinese compound word is visually presented, the semantic representations of both the entire word and the constituent characters are considered (Zhou and Marslen-Wilson, 1994, 1995). Thus, readers need to solve ambiguity at the character (sublexical) level using information derived from the other constituent characters and the whole word, termed “sublexical ambiguity resolution” (Huang C. Y. et al., 2011; Huang H. W. et al., 2011; Huang and Lee, 2018). Studies of Chinese word recognition in young adults have suggested that semantically unrelated morphemes are represented as separate entries (Huang H. W. et al., 2011; Huang and Lee, 2018). When retrieving the meaning of a sublexically ambiguous word, a competitive process occurs between multiple meanings. And thus, results have demonstrated a sublexical ambiguity disadvantage effect in reading (Huang H. W. et al., 2011; Huang and Lee, 2018)—words with multiple meanings at the sublexical level delay word recognition relative to words with one meaning. However, research into the effects of aging on Chinese word recognition remains limited.

Healthy older adults experience a general decline in physical and cognitive abilities with age. Yet, the rates of decline in behavioral and neurocognitive abilities have been shown to be highly variable within the older population (Li, 2003; Goh et al., 2012; Fan et al., 2019), highlighting the importance of considering such individual differences when studying aging populations. In addition to vocabulary, category verbal fluency, and processing speed, factors typically considered to influence word recognition performance (Shao et al., 2014), education has been suggested by the neurocognitive reserve hypothesis as a protector against the negative effect of aging (Stern, 2009; Cabeza et al., 2018; Huang and Huang, 2019). Many studies on the older population have observed the facilitatory impact of education on various language abilities, ranging from the lexical to the sentence level. At the lexical level, individuals with higher education perform better in picture naming (Inouye et al., 1993; Acevedo et al., 2007; Welsh-Bohmer et al., 2009; Constantinidou et al., 2012), demonstrate better word knowledge (Denney and Thissen, 1983; Inouye et al., 1993; Barnes et al., 2004; Acevedo et al., 2007; Welsh-Bohmer et al., 2009; Constantinidou et al., 2012), and show a smaller frequency effect in word recognition (Tainturier et al., 1992) than less educated individuals. At the sentence level, highly educated individuals respond to sentence stimuli more accurately (Mungas et al., 2005; Carvalho et al., 2009; Ferreira et al., 2015) than their less educated counterparts.

Perceived loneliness, or the perception of being socially isolated (Hawkley and Cacioppo, 2010), has been identified as a significant concern among elderly populations (Cacioppo et al., 2006, 2010). Forty percent of adults over 65 years of age report being lonely at least occasionally, and this number increases with age (Pinquart and Sorensen, 2001). The sudden COVID-19 outbreak and the prolonged pandemic that followed severely disrupted normal human social interactions and cultivated a heightened sense of loneliness (Kokou-Kpolou et al., 2020; Voitsidis et al., 2020; Wu, 2020). Research has shown that greater perceived loneliness is associated with reduced cognitive function in aging populations, particularly in terms of working memory and processing speed (Boss et al., 2015). A negative correlation has also been reported between perceived loneliness and executive function (e.g., planning; Sin et al., 2021). Moreover, studies have reported a negative relationship between loneliness and verbal fluency (Schnittger et al., 2012), suggesting that a decrease in daily social interactions reduces language processing abilities. However, whether perceived loneliness influences ambiguity resolution in older adults has not yet been explored.

Therefore, to examine the effects of aging and loneliness on word recognition, we adapted the materials and procedures from Huang and Lee (2018) for use with neurologically normal older adult participants aged between 60 and 80. A lexical decision task (i.e., differentiating words versus pseudowords) was used, allowing us to differentiate age-related differences at both the word (lexicality effect) and sublexical (sublexical ambiguity effect) levels. Specifically, the lexicality effect refers to differences in response time and accuracy when differentiating between words and pseudowords. In contrast, the sublexical ambiguity effect refers to such differences between sublexically ambiguous words and sublexically unambiguous words. Given the general decline in physical and cognitive abilities that have been observed with age, we hypothesize that both the lexicality and sublexical ambiguity effects increase in older adults compared with younger adults. We then examined whether individual variations in word recognition and resolution of sublexical ambiguity are driven by differences in verbal abilities, processing speeds, or education levels among the elderly population. Finally, we examined whether perceived loneliness negatively impacts word recognition and sublexical ambiguity resolution.

## Materials and methods

### Participants

Fifty healthy, right-handed older adults participated in this study (for their demographic information, see Table 1). They were recruited from the local community. All of the participants were native Chinese speakers. The participants’ psychological conditions were assessed using the Montreal Cognitive Assessment (MoCA; Nasreddine et al., 2005) and Geriatric Depression Scale (GDS; Yesavage et al., 1983). The participants who had a cognitive condition no worse than “mildly impaired” (i.e., scoring higher than 18 points in the MoCA, which is the cutoff point between “mild cognitive impairment” and “moderate cognitive impairment”) and depression no worse than “mildly depressive” (i.e., scoring lower than 19 points in the GDS, which is the cutoff point between “mildly depressed” and “severely depressed”) were included in the study. All participants passed the cognitive screening. This study was approved by the Institutional Review Board of Academia Sinica, Taiwan.

**Table 1:** Participant characteristics.

Measure	Young (*N* = 38)	Older (*N* = 50)
Age (years)	22.21 (2.51)	66.38 (4.86)
Sex	20/38 women	36/50 women
Length of education (years)	—	14.62 (3.12)
Montreal Cognitive Assessment	—	24.66 (2.66)
Geriatric Depression Scale	—	6.12 (4.28)
WAIS-III (vocabulary)	—	49.56 (7.82)
Category verbal fluency	—	17.31 (2.23)
WAIS-III (digit-symbol coding)	—	68.68 (14.27)
UCLA Loneliness Scale	—	38.54 (8.22)

Means and standard deviations are reported. The standard deviation values are in parentheses.

Data from young participants were extracted from Huang and Lee (2018).

WAIS-III, Wechsler Adult Intelligence Scale, Third Edition.

### Lexical decision task

One hundred and twenty Chinese disyllabic compound words (i.e., words whose meanings are contributed to by the meanings of their constituent characters) were selected as experimental stimuli from the Academia Sinica balanced corpus (Huang and Chen, 1998). As an indicator of sublexical ambiguity, these words were evenly divided into two subsets according to the number of meanings (NOM) of their first character (one vs. multiple meanings; the NOM of the multiple-meaning subset ranged from 2 to 7). For example, 光腳 (kuang1 jiao3, *barefoot*) is a sublexically ambiguous word, whereby the first character 光 (kuang1) has at least three meanings (*light*, *naked*, and *simply*). In contrast, 糖漿 (tang2 jiang1, *syrup*) is a sublexically unambiguous word, as the first character 糖 (tang2) has only one meaning (*sugar*). The NOMs were collected from the Academia Sinica Chinese Wordnet. Potential confounding factors, including word frequency (WF) and orthographic neighborhood size^1^ of the first character (NS1), orthographic neighborhood size of the second character (NS2)^2^, and the NOMs corresponding to the second character were controlled (see Table 2). One hundred and twenty pseudowords were created by concatenating two real characters in such a way that the generated combinations did not appear in the word corpus or resemble real words in pronunciation. A list containing 10 real words and 10 pseudowords was constructed for the practice session.

**Table 2:** Descriptive statistics of the lexical decision task.

Category	NOM	WF	NS1	NS2
NOM = 1	1.00	16.05	21.95	16.15
NOM > 1	3.05	16.20	23.75	21.55

Means are reported.

NOM, number of meanings; WF, word frequency; NS1, orthographic neighborhood size of the first character; NS2, orthographic neighborhood size of the second character.

### Neuropsychological tests

A range of neuropsychological tests were used to assess the participants’ verbal abilities, processing speeds, and perceived loneliness. Verbal ability was assessed using the vocabulary subtest of the Wechsler Adult Intelligence Scale-III (WAIS-III; Wechsler, 1997) and a category verbal fluency test (Chung et al., 2007). In the vocabulary test, the participants were asked to provide definitions for 33 words presented to them consecutively. The test would be terminated if a participant could not define six consecutive words. The score was calculated based on the number of correctly defined words and the quality of the definitions. In the category verbal fluency test, the participants were asked to produce as many words as possible in 1 min belonging to the categories of animals, fruits, colors, and place names in Taiwan, in separate sessions. The score was the number of correct and non-repeated words.

Processing speed was assessed with the WAIS-III digit-symbol coding subtest (Wechsler, 1997). In this test, the worksheet contained blank squares each paired with a number ranging from 1 to 9. Above these squares was a key row matching each number with a unique symbol. The participants were asked to write the symbol corresponding to the number associated with each blank square within 2 min.

Perceived loneliness was measured using the 20-item University of California, Los Angeles (UCLA) Loneliness Scale (Russell et al., 1980; Liu, 1993). This scale uses a 4-point Likert scale (1 = never to 4 = always), with higher scores indicating a higher level of perceived loneliness.

### Procedure

A demographic questionnaire was used to collect the participants’ age, job and length of education. Then, a set of neuropsychological tests and a lexical decision task were completed consecutively with intermissions.

For the lexical decision task, the participants were seated in front of a monitor in a soundproof room. They were instructed to put their index finger and middle finger on two designated buttons of a response box and look at the displayed words for comprehension and make a lexical decision (i.e., decide whether the displayed word was a real word or pseudoword, and press the corresponding button on the response box to indicate their decision) as quickly and accurately as possible.

Before each trial, a white plus sign appeared in the center of the monitor for 500 ms. The participants were instructed to focus on the plus sign and minimize blinking and eye movements. A blank screen was then displayed for a random period between 300 and 700 ms. Next, a word was displayed for the participants to make a lexical decision by pressing the button under their index or middle finger to indicate a real word or pseudoword, respectively. This display ended when the participants pressed a button or after 2000 ms if the participants made no judgment. After the word disappeared, a blank screen was displayed such that the total display time of the word and the blank screen was 2000 ms. The intertrial interval was 2,500 ms.

The experimental part contained 240 randomized trials and was evenly divided into four blocks. The participants spent around 5 min on each block and took a short break between blocks. Before the experimental trials, the participants completed a short practice session containing 20 trials to familiarize themselves with the task.

### Statistical analysis

To examine the effect of aging, data from Huang and Lee (2018) describing 38 young adults were extracted and the performance of the older adults in this study were compared (for demographic information, see Table 1). These young adults carried out the same lexical decision task as in the present study. All of the participants were right-handed undergraduate students and native Chinese speakers. Mixed multi-factorial analysis of variance (ANOVA) was used to examine the effect of aging on the lexicality and sublexical ambiguity effects. These analyses each included two ANOVAs, with each ANOVA using either accuracy or reaction time in the lexical decision task as the dependent variable. Only the data corresponding to correct responses were included in the analyses. In terms of the lexicality effect, the two ANOVAs used age (older vs. young) as the between-subjects independent variable and lexicality (real word vs. pseudoword) as the within-subjects independent variable. Each participant’s performance was averaged within each level of lexicality. The two ANOVAs for the sublexical ambiguity effect used age (older vs. young) as the between-subjects independent variable and sublexical ambiguity (unambiguous (NOM = 1) vs. ambiguous (NOM > 1)) as the within-subjects independent variable. Only the data corresponding to real words were used, and each participant’s performance was averaged within each level of sublexical ambiguity. In cases where the interaction effect was significant, paired t-tests were conducted for each age group to determine whether the within-subjects variable varied significantly with age.

To assess the influence of individual differences such as education, verbal abilities, processing speed, and perceived loneliness on word recognition and resolution of sublexical ambiguity among older adults, multiple linear regression analyses were conducted using the length of education (ED), vocabulary test scores (VC), category verbal fluency test (VF), digit-symbol coding test (DSC), and UCLA Loneliness Scale (LS) as predictors. As higher education has become more desirable and valued in job recruitment in recent decades, a correlation between age and length of education may exist (i.e., younger people have received more education), potentially making age a confounding factor when examining the effects of education. Therefore, age was included as an additional predictor when constructing the regression models to allow us to examine whether the potential effects of education can be accounted for by age alone. Three dependent variables were incorporated to reflect word recognition performance: reaction time to real words, reaction time to pseudowords, and the sublexical ambiguity effect (the difference in reaction times to sublexically ambiguous words (NOM > 1) and sublexically unambiguous words (NOM = 1)). For each dependent variable, a multiple linear regression analysis was carried out using the forward hierarchical method. In this method, the predictor with the strongest correlation with the dependent variable enters the model first, and a newly entered predictor is retained if it adds significantly to the model. Otherwise, the predictor is excluded. Model comparisons were done using the *anova* function in *r*.

## Results

### Age-related differences in lexicality and sublexical ambiguity

When examining how aging affects the lexicality effect, a mixed multi-factorial ANOVA using accuracy as the dependent variable (see Table 3) indicated a significant between-subjects difference in age (*F* (1,86) = 25.748, *p* < 0.001), with older adults’ accuracy being significantly lower than that of younger adults. Within-subjects differences in lexicality were also significant (*F* (1,86) = 38.643, *p* < 0.001), with the accuracy of the participants’ responses to real words being significantly higher than those for pseudowords. Moreover, a significant interaction was seen between age and lexicality (*F* (1,86) = 31.124, *p* < 0.001). Further examination using *post hoc* tests for each age group showed a significant lexicality effect in older adults (*t* (49) = −6.91, *p* < 0.001), while this was negligible in younger adults (*t* (37) = −1.40, *p* > 0.05). The mixed multi-factorial ANOVA using reaction time as the dependent variable revealed similar results (see Table 3). The main effects of age and lexicality were significant (*F* (1,86) = 293.939, *p* < 0.001; *F* (1,86) = 231.732, *p* < 0.001, respectively), with older adults reacting more slowly and pseudowords prompting slower responses. The interaction between age and lexicality was also significant (*F* (1,86) = 105.667, *p* < 0.001). Further examination within each age group revealed significant lexicality effects for both age groups. However, the effect size (measured by Cohen’s *d*) in older adults was larger than that in younger adults (*t* (49) = 15.1, *p* < 0.001, *d* = 2.14; *t* (38) = 8.56, *p* < 0.001, *d* = 1.39, respectively).

**Table 3:** Descriptive statistics of participants’ lexical decision performance, grouped by age and lexicality.

Lexicality	Young		Older	
	Accuracy	Reaction time (ms)	Accuracy	Reaction time (ms)
Real word	0.96 (0.02)	627.09 (84.93)	0.91 (0.05)	921.16 (132.07)
Pseudoword	0.95 (0.06)	701.74 (92.35)	0.73 (0.24)	1306.28 (200.01)

Means and standard deviations are reported. The standard deviation values are in parentheses.

To examine how aging modulates the sublexical ambiguity effect, a mixed multi-factorial ANOVA using accuracy as the dependent variable (see Table 4) was used and showed that neither age, sublexical ambiguity, nor their interaction significantly affected accuracy (*F*(1,86) = 1.558, *p* > 0.05; *F*(1,86) = 0.064, *p* > 0.05; *F*(1,86) = 0.973, *p* > 0.05, respectively). In contrast, a mixed multi-factorial ANOVA using reaction time as the dependent variable (see Table 4) revealed significant effects of age and sublexical ambiguity (*F*(1,86) = 143.078, *p* < 0.001; *F*(1,86) = 19.937, *p* < 0.001, respectively), with older adults reacting significantly slower and sublexically ambiguous words prompting slower responses. The interaction between age and sublexical ambiguity was not significant (*F*(1,86) = 2.247, *p* > 0.05).

**Table 4:** Descriptive statistics of participants’ lexical decision performance, grouped by age and sublexical ambiguity (i.e., NOM).

NOM	Young		Older	
	**Accuracy**	**Reaction time (ms)**	**Accuracy**	**Reaction time (ms)**
= 1	0.96 (0.02)	621.85 (80.33)	0.97 (0.05)	911.04 (132.29)
> 1	0.96 (0.03)	631.80 (91.51)	0.97 (0.05)	931.03 (134.18)

Means and standard deviations are reported. The standard deviation values are in parentheses.

NOM, number of meanings.

### Individual differences in word recognition and resolution of sublexical ambiguity among older adults

To explore the effects of individual differences on word recognition, linear regression models were constructed to predict reaction times to real words and pseudowords. The resulting significant linear regression model in predicting reaction times to real words (*F*(1,48) = 17.87, *p* < 0.001, *R*^2^ = 0.271, Adj. *R*^2^ = 0.256) included only DSC as a predictor (*β* = −4.822, *t* = −4.228, *p* < 0.001). Interestingly, the inclusion of additional predictors did not significantly improve model performance. Similarly, the significant model in predicting reaction times to pseudowords (*F*(1,48) = 8.812, *p* < 0.01, *R*^2^ = 0.156, Adj. *R*^2^ = 0.138) also included DSC as its only predictor (*β* = −5.521, *t* = −2.968, *p* < 0.01). These results suggest that a faster processing speed (i.e., better DSC score) can predict the speed of recognition of real words and rejection of pseudowords.

We similarly constructed a linear regression model to explore the effects of individual differences on sublexical ambiguity resolution. The resulting significant linear regression model in predicting the sublexical ambiguity effect (*F*(1,48) = 4.483, *p* < 0.05, *R*^2^ = 0.085, Adj. *R*^2^ = 0.067) only included LS as a predictor (*β* = 1.179, *t* = 2.117, *p* < 0.05). As such, the higher the loneliness score, the greater the sublexical ambiguity effect. This result suggests that loneliness significantly impacts efficacy in resolving sublexical ambiguity when reading.

## Discussion

### Visual word recognition across the human lifespan

At the word level, the results showed lexicality effects were significant in both younger and older populations. However, this effect was more pronounced in older adults. Greater lexicality effects in older adults may relate to the prolonged cognitive processes involved in discriminating words from pseudowords, as opposed to ambiguity resolution itself. This age-related difference is consistent with the common finding that older adults rely more on top-down lexical information during word recognition (Cohen-Shikora and Balota, 2016). Reading real words activates the word-level representations of these words in the mental lexicon, whereas reading pseudowords does not, as pseudowords do not have such representations. This word-level representation provides older adults with top-down information to aid the activation and recognition of constituent characters, improving their ability to recognize real words, in contrast to rejecting pseudowords, leading to a greater lexical effect. Our results are consistent with previous empirical findings from spoken word recognition: when older adults heard only the initial part of a word without any context, their recognition of this word was less accurate than young adults. However, providing context could narrow these differences between age groups (Wingfield et al., 1991).

At the sublexical level, ambiguity effects were similar among younger and older adults. Based on the behavioral results, this finding may suggest that older readers access the semantic representations of Chinese compound words through the morphemic representations of their constituent characters in a similar way to younger adults. When a constituent character has multiple meanings, the mapping of its orthographic to semantic representation is one-to-many, leading to the activation of multiple morphemic representations. This activation and the subsequent selection process cause a delay in word recognition. Initially, our observation of no significant age-related differences in the sublexical ambiguity effect appeared inconsistent with the results of previous studies (Faust et al., 1997). However, most studies have addressed ambiguity effects at the word level rather than at the sublexical level. The similarity in the sublexical ambiguity effect found between the two age groups indicates that older adults (as a group) can use whole word information efficiently to select the appropriate meaning for words containing an ambiguous first character. This overall pattern suggests that age-related changes in ambiguity resolution do not extend to the sublexical level.

### Individual differences among older adults

When individual differences were considered among the older participants, predictors of visual word recognition at the word level and the sublexical level were significantly different. For real word recognition and pseudoword rejection, the only significant predictor was processing speed. In contrast, for sublexical ambiguity resolution, the only significant predictor was perceived loneliness.

Our finding that faster processing speed robustly predicts faster general word recognition (i.e., real word recognition and pseudoword rejection) is consistent with the findings of previous studies showing that processing speed accounts for variations in reaction times across a wide range of cognitive tasks (Verhaeghen and Salthouse, 1997; Yap et al., 2012), including age-related declines in language performance and processing (Kwong See and Ryan, 1995). It should be noted that although verbal abilities such as vocabulary and category verbal fluency did not significantly predict the speed of word recognition in our models, this does not mean that they are unrelated to word recognition. As our models were constructed with a forward hierarchical method, they only included predictors with independent contributions. Verbal abilities have long been known to be associated with word recognition performance, and this relationship would be evident if processing speed was controlled for prior to model construction (Yap et al., 2012; Shao et al., 2014).

It was surprising that education did not significantly predict general word recognition performance or sublexical ambiguity resolution, despite it being reported as a protector against the negative effects of aging (Stern, 2009; Cabeza et al., 2018; Huang and Huang, 2019) and a good predictor of various language functions (Denney and Thissen, 1983; Tainturier et al., 1992; Inouye et al., 1993; Barnes et al., 2004; Mungas et al., 2005; Acevedo et al., 2007; Carvalho et al., 2009; Welsh-Bohmer et al., 2009; Constantinidou et al., 2012; Ferreira et al., 2015). One possible explanation for this result may be that the length of education in our study (14.6 years on average) was slightly higher than in most previous studies examining the effect of education on language functions (Table 5). The reason may be that basic education in Taiwan was set at 6 years in 1943, while the 9-year compulsory education system was implemented in 1968. Furthermore, research has shown that children with 6 years of education in Taiwan can recognize 66% of high-frequency characters (Wang et al., 2008). Although we found no studies assessing children’s literacy several decades ago, the constituent characters of our word stimuli were of high frequency, and most of the word stimuli in the present research were likely to be familiar to our participants, which could explain why education was not a reliable predictor of word recognition in general and was not a sensitive measure for disentangling individual differences in the use of whole word knowledge while resolving sublexical ambiguity.

**Table 5:** Descriptive statistics of length of education in previous studies.

Articles	*N*	Years of education
Verhaeghen (2003)	321	15.0 (1.4)
Acevedo et al. (2007)	89	11.9 (3.8)
Barnes et al. (2004)	664	15.0 (3.0)
Constantinidou et al. (2012)	231 (younger)	8.3 (4.3)
	128 (older)	7.2 (3.8)
Welsh-Bohmer et al. (2009)	507	13.4
Tainturier et al. (1992)	10 (high education level)	18.0
	10 (low education level)	10.7
Mungas et al. (2005)	527	9.3 (5.8)
The present study	50	14.6 (3.12)

In the “Years of Education” column, means, and standard deviations are reported. The standard deviation values are in parentheses.

As education was not included in any of our regression models because of its insufficient independent contribution, we could not determine whether the potential effects of education were linked to age. However, the correlation between age and length of education among our participants was not significant (*r* = 0.05, *p* > 0.05), indicating that age is not likely to account for the effect of education on word recognition performance. This low correlation may be attributed to the age range of our participants, which was not wide enough to capture the changes in educational attainment over time.

### The impact of perceived loneliness on visual word recognition

As for the level of perceived loneliness, it had no impact on the general response times for word recognition or pseudoword rejection. It did, however, uniquely modulate the sublexical ambiguity effect. The non-significant effect of processing speed on sublexical ambiguity effect suggests that it affects the recognition of words with different levels of ambiguity to a similar degree. When reading a sublexically ambiguous Chinese word, readers need to consider the meaning of both characters, form a coherent whole word meaning, and select the most appropriate meaning of the first character. Our results may suggest that a lack of social connection can harm individuals’ ability to establish semantic connections between constituent characters in a word and, in turn, hinder their selection of an appropriate meaning for the first character. Alternatively, our findings may suggest that insufficient social connections limit language use and may harm an individual’s ability to inhibit irrelevant linguistic information. This view is consistent with the finding of Cacioppo et al. (2000) that lonely individuals display deficits in voluntary control of their attention and inhibition of distracting information. In their dichotic listening task in which the participants were asked to focus on the information presented to their subdominant ears, while the control participants made more correct responses to the information presented to their subdominant ears than to their dominant ears, the lonely participants’ accuracy on the two kinds of information was not significantly different.

Perceived loneliness has been shown to have negative effects on the planning and working memory components of executive function in older adults (Sin et al., 2021); however, studies of its influence on other components of executive function relating to language processing (such as inhibition control) are scarce and their findings are inconclusive (O’Luanaigh et al., 2012; Schnittger et al., 2012; Shankar et al., 2013; Sin et al., 2021). Although perceived loneliness was shown to impact the efficiency of sublexical ambiguity resolution, indicating that it may affect the use of contextual information and inhibition processes in reading Chinese, the present study could not provide evidence on whether perceived loneliness has an impact on meaning activation or selection in ambiguity resolution. Given the prominent role of language in social interaction, more studies are needed to examine how social isolation and perceived loneliness impact language processing and its neural mechanisms.

### Strengths and limitations

Our results showed that loneliness can have an adverse effect on visual word recognition. This finding advances our understanding of the role played by social factors in the cognitive vitality of older adults and underscores the importance of maintaining older adults’ mental health. Older adults undergo transitions in their social lives after retirement or bereavement, which in many cases lead to social isolation and loneliness. Most recently, quarantine during the global COVID-19 outbreak led to prolonged social isolation, which may increase feelings of loneliness; therefore, loneliness is a critical public health concern that must be considered when using social isolation to combat the spread of the virus (Heidinger and Richter, 2020).

In our multiple linear regression analyses, we examined five predictors. According to Tabachnick et al. (2007), for such analyses the sample size should be at least 50 plus an additional eight participants per predictor. This suggests that our sample size of 50 was insufficient. To account for this, we used a forward hierarchical method when constructing our linear regression models so as to only include predictors with significant independent contributions. This means that the resulting models were parsimonious in terms of the number of predictors included. To check whether the power of these models was adequate (i.e., above 0.8) with our current sample size (*N* = 50), we used G*Power (Faul et al., 2009). The power of our models in predicting reaction times to real words and pseudowords was adequate (0.99 and 0.84, respectively). However, the power of the model predicting the sublexical ambiguity effect was inadequate (0.56). With the current effect size of the predictor (LS in this case), we would need 86 participants to obtain a model with adequate power. Further work involving a larger study population is thus needed to construct a more accurate model for predicting sublexical ambiguity.

## Conclusion

Overall, this study demonstrated that older adults as a group show a greater lexicality effect and a similar sublexical ambiguity effect in reading Chinese words when compared with young adults. We found that individual characteristics can influence basic visual word recognition, such that processing speed affects general word recognition times, but does not appear to affect the sublexical ambiguity resolution of Chinese words. The negative association between the level of perceived loneliness and the efficiency of sublexical ambiguity resolution found in the current study provides an important link between subjective distress and lexical processing, highlighting the importance raising awareness of the negative effects of loneliness in older adults, especially during the global COVID-19 pandemic, which led to prolonged social isolation.

## Data availability statement

The raw data supporting the conclusions of this article will be made available by the authors, without undue reservation.

## Ethics statement

The studies involving human participants were reviewed and approved by Institutional Review Board of Academia Sinica, Taiwan. The patients/participants provided their written informed consent to participate in this study.

## Author contributions

NZ analyzed, interpreted the data, and wrote the paper. C-MH allocated the funding, designed the study, interpreted the data, and wrote the manuscript. QC interpreted the data, and wrote the manuscript. OT allocated the funding, and interpreted the data. H-WH allocated the funding, designed the study, collected, analyzed, interpreted the data, and wrote the manuscript. All authors contributed to the article and approved the submitted version.

## Funding

This work was supported by Academia Sinica’s Thematic Research Program (AS-103-TP-C04) (for C-MH, H-WH, and OT), by the Center for Intelligent Drug Systems and Smart Bio-Devices (IDS^2^B) from The Featured Areas Research Center Program within the framework of the Higher Education Sprout Project by the Ministry of Education (MOE) in Taiwan, and by Taiwan’s Ministry of Science and Technology (105-2420-H-009-001-MY2; 106-2410-H-001-024-MY2; 107-2410-H-009-028-MY3) (for C-MH). This work was also supported by City University of Hong Kong (7005343; 7005414) (for H-WH).

## Conflict of interest

The authors declare that the research was conducted in the absence of any commercial or financial relationships that could be construed as a potential conflict of interest.

## Publisher’s note

All claims expressed in this article are solely those of the authors and do not necessarily represent those of their affiliated organizations, or those of the publisher, the editors and the reviewers. Any product that may be evaluated in this article, or claim that may be made by its manufacturer, is not guaranteed or endorsed by the publisher.

## Footnotes

^1^Orthographic neighborhood size is the number of words that share the same constituent character at the same position. For example, 花園 (hua1 yuan2, *flower garden*) is an orthographic neighbor of 花市 (hua1 shi4, *flower market*), sharing the first character, and 都市 (du1 shi4, *city*) is an orthographic neighbor of 花市 (hua1 shi4, *flower market*), sharing the second character.

^2^Although previous literature has suggested that the first constituent character plays a much more important role than the second one in reading Chinese words (Huang et al., 2006), we still try to match the orthographic neighborhood size of the second character. However, the NS2 for sublexically unambiguous condition (NOM = 1) is slightly smaller than that for sublexcially ambiguous condition (NOM > 1) in the stimulus set.
